# Insulin Sensitizing Effects of Oligomannuronate-Chromium (III) Complexes in C2C12 Skeletal Muscle Cells

**DOI:** 10.1371/journal.pone.0024598

**Published:** 2011-09-15

**Authors:** Cui Hao, Jiejie Hao, Wei Wang, Zhangrun Han, Guangsheng Li, Lijuan Zhang, Xia Zhao, Guangli Yu

**Affiliations:** 1 Key Laboratory of Marine Drugs, Chinese Ministry of Education, Ocean University of China, Qingdao, People's Republic of China; 2 Shandong Provincial Key Laboratory of Glycoscience and Glycotechnology, Ocean University of China, Qingdao, People's Republic of China; Pennington Biomedical Research Center, United States of America

## Abstract

**Background:**

It was known that the insulin resistance in skeletal muscle is a major pathogenic factor in diabetes mellitus. Therefore prevention of metabolic disorder caused by insulin resistance and improvement of insulin sensitivity are very important for the therapy of type 2 diabetes. In the present study, we investigated the ability of marine oligosaccharides oligomannuronate and its chromium (III) complexes from brown alga to enhance insulin sensitivity in C2C12 skeletal muscle cells.

**Methodology/Principal Findings:**

We demonstrated that oligomannuronate, especially its chromium (III) complexes, enhanced insulin-stimulated glucose uptake and increased the mRNA expression of glucose transporter 4 (GLUT4) and insulin receptor (IR) after their internalization into C2C12 skeletal muscle cells. Additionally, oligosaccharides treatment also significantly enhanced the phosphorylation of proteins involved in both AMP activated protein kinase (AMPK)/acetyl-CoA carboxylase (ACC) and phosphoinositide 3-kinase (PI3K)/protein kinase B (Akt) signaling pathways in C2C12 cells, indicating that the oligosaccharides activated both the insulin signal pathway and AMPK pathways as their mode of action. Moreover, oligosaccharides distributed to the mitochondria after internalization into C2C12 cells and increased the expression of transcriptional regulator peroxisome proliferator-activated receptor γ coactivator-1α (PGC-1α), carnitine palmitoyl transferase-1 (CPT-1), and phosphorylated acetyl-CoA carboxylase (p-ACC), which suggested that the actions of these oligosaccharides might be associated with mitochondria through increasing energy expenditure. All of these effects of marine oligosaccharides were comparable to that of the established anti-diabetic drug, metformin. In addition, the treatment with oligosaccharides showed less toxicity than that of metformin.

**Conclusions/Significance:**

Our findings indicate that oligomannuonate and its chromium (III) complexes improved insulin sensitivity in C2C12 skeletal muscle cells, and acted as a novel glucose uptake stimulator with low toxicity, and could be used as dietary supplementary or potential drug for type 2 diabetes mellitus.

## Introduction

Diabetes mellitus is the most common metabolic disease and its prevalence is increasing in both developed and developing countries. More than 90% of diabetes patients suffer from non-insulin-dependent diabetes mellitus (NIDDM, type 2 diabetes) [1]. Type 2 diabetes is associated with two principal physiological defects: resistance to the action of insulin and deficiency in insulin secretion [2]. Insulin resistance in skeletal muscle is a major pathogenic factor in type 2 diabetes mellitus [3].

Previous studies have indicated that glucose transport is the rate-limiting step for glucose metabolism in skeletal muscle [4], a major site of glucose disposal during the insulin-stimulated state in vivo [5]. GLUT4 is the main insulin-responsive glucose transporter and is expressed primarily in skeletal and cardiac muscle tissues [6], [7]. AMPK is an important enzyme in the regulation of cellular energy status and plays a key role in regulating plasma glucose levels [8]. In skeletal muscle, AMPK is activated during contraction that leads to recruitment of GLUT4 to the plasma membrane [9]. It is now known that metformin (dimethylbiguanide) can activate AMPK and that, at least in part, explains its anti-diabetic activity [10]. Gastrointestinal side effects are the most common adverse events of metformin, occurring in 20∼30% of patients [11]. Therefore, an alternative anti-diabetic drug with low toxicity and side effect is needed.

In recent years, lots of poly-/oligosaccharides have been investigated and showed antidiabetic activities [12]. For example, hypoglycemic activities have been demonstrated for chito-oligosaccharides and its derivatives [13], oligosaccharides from *Amorphophallus konjac* [14] and *Rehmannia glutinosa* [15]. In addition, the recognized role of chromium (III) in glucose homeostasis has lead to the investigation of chromium (III) complexes, especially oligosaccharides-chromium (III) complexes, for use as insulin-enhancing approaches for the treatment of type 2 diabetes mellitus [16], [17].

Marine brown algae contain a wide variety of acidic polysaccharides such as the alginate and the fucoidans. Alginate, a water-soluble linear polymer, is an anionic heteropolysaccharide comprised of β-D-mannuronic acid (M) and α-L-guluronic acid (G) [18]. Alginate oligosaccharides have attracted lots of scientific interest owning to their various biological activities, such as promoting root growth in higher plants [19], [20], antitumor [21], [22], and neuron protection effects [23], [24]. Moreover, some alginate-derived oligosaccharide and its sulfate showed a better anti-diabetes activity [25], [26].

In the present work, we prepared oligomannuronate (OM) and two kinds of oligomannuronate-chromium (III) complexes (OM2 and OM4) from marine brown alga *Laminaria japonica*, and their insulin sensitizing effects in C2C12 skeletal muscle cells were studied. The results showed that all oligosaccharides, especially oligomannuronate-chromium (III) complex OM2 could enhance glucose uptake in the C2C12 cells without obvious toxicity. The improvement effect might be attributed to the upregulated expression of IR mRNA and GLUT4 mRNA levels by activating both PI3K/Akt and AMPK pathways. Moreover, those oligosaccharides also distributed to the mitochondria in C2C12 cells and increased the expression of PGC-1α and CPT-1, which suggested the actions of these oligosaccharides might be associated with mitochondria. Therefore, the oligomannuronate-chromium (III) complexes could be used as potential anti-diabetes drugs for improving the insulin sensitivity.

## Results

### Enhancement of glucose uptake by oligomannuronate and its chromium (III) complexes

**Figure 1:**
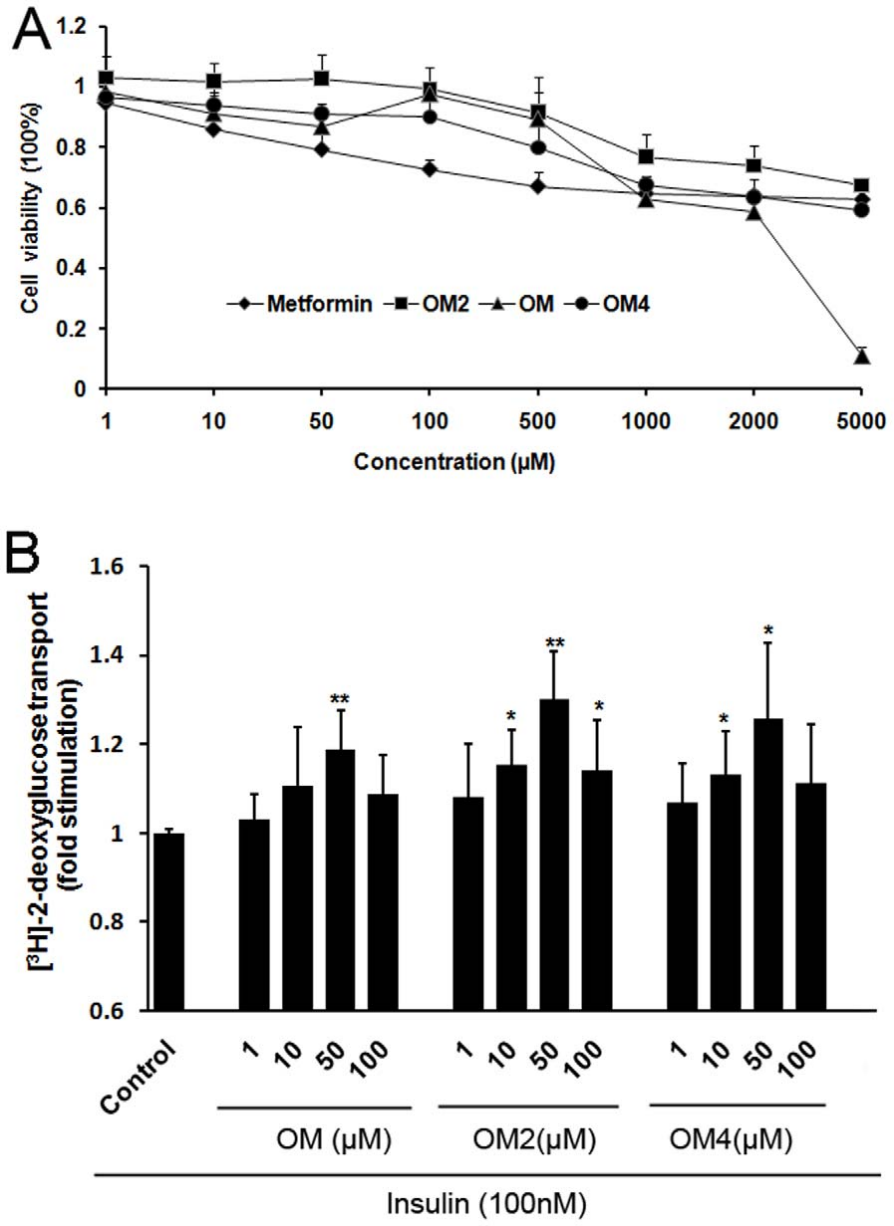
The cytotoxicity and effects of compounds on insulin stimulated glucose uptake in myoblasts C2C12 cells. (A) C2C12 cells were incubated with oligomannuronate and its chromium (III) complexes (OM, OM2, OM4) at indicated concentrations for 24 h. The cell viability was evaluated by MTT assay as described in materials and methods. Values are mean ± SD of four replicates. (B) C2C12 cells were treated with or without different concentrations of indicated compounds for 24 h. Then after washed with PBS, cells were stimulated with insulin for 30 minutes before performing glucose transport assay. The radioactivity amount for untreated cells (control) were assigned values of 1 and the results presented as mean ± S.D. (n = 4). Significance: *P<0.05 and **P<0.01 versus controls without treatment.

Prior to evaluate the effects of oligomannuronate and its chromium (III) complexes on glucose uptake in C2C12 cells, their cytotoxicity profiles were determined by MTT assay. Each of the oligomannuronate and its chromium (III) complexes showed no significant cytotoxicity up to 500 µM (Figure 1A), but in contrast, metformin demonstrated milder cytotoxic at 100 µM. Interestingly, both OM2 and OM4 showed higher cell viability than OM at the same concentrations, and this was particularly pronounced at the concentration of 5000 µM (Figure 1A).

In order to examine whether oligomannuronate and its chromium (III) complexes could affect insulin stimulated-glucose uptake in mouse C2C12 skeletal muscle cells or not, 100 nM insulin were added to the oligosaccharides treated group, and their glucose uptake were compared with the non-insulin treated group (control). As shown in Figure 1B, the marine acidic oligosaccharide OM, and its chromium (III) complexes OM2 and OM4 apparently increased glucose uptake at the concentrations from 1 to 100 µM. Compared with the untreated (control) group, OM increased 20% insulin stimulated-glucose uptake, whereas OM2 and OM4 increased the insulin-stimulated glucose uptake by 30% and 25% at 50 µM, respectively (Figure 1B). Moreover, as shown in Figure 1B, the most effective concentration of oligomannuronate and its chromium (III) complexes to enhance insulin stimulated glucose uptake was 50 µM, and OM2 was the most effective compound among the oligosaccharides.

### Effects of oligomannuronate and its chromium (III) complexes on the mRNA expression of IR and GLUT4

**Figure 2:**
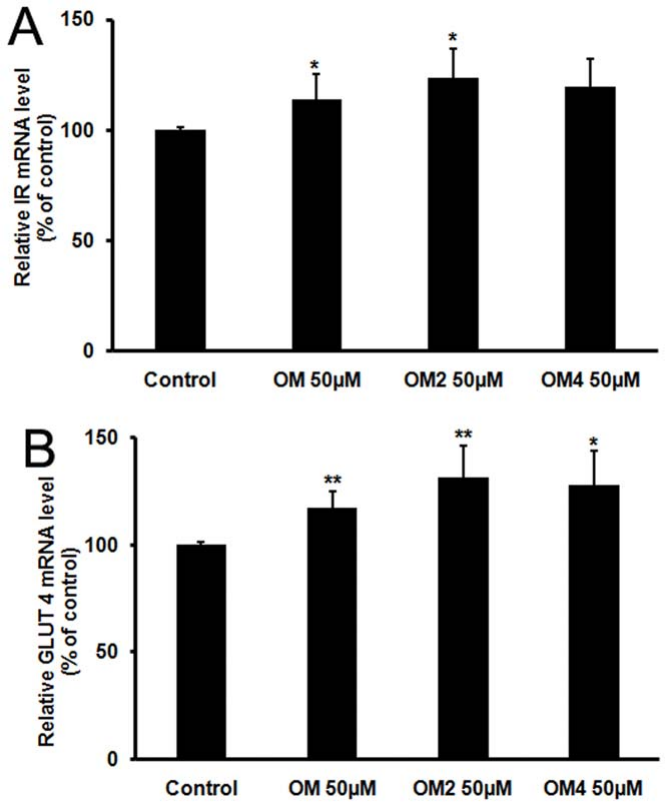
Effects of oligosaccharides treatments on the mRNA expression of IR and GLUT4 in C2C12 cells. Skeletal muscle cells were left untreated or treated with 50 µM oligomannuronate and its chromium (III) complexes (OM, OM2, OM4) in DMEM media for 24 h. The mRNA levels of IR (A) and GLUT4 (B) were analyzed by RT-PCR as described in the text. The mRNA levels for untreated cells (control) were assigned values of 100. Values are mean ± SD of the results from at least four independent experiments. Significance: *P<0.05, **P<0.01 versus controls.

GLUT4 is the main insulin-responsive glucose transporter in skeletal muscle [27]. The expression levels of IR and GLUT4 were quantitatively analyzed in C2C12 cells by real time RT-PCR. The C2C12 cells were incubated in the media containing 50 µM oligosaccharides (OM, OM2, and OM4) for 24 h. Total RNA was extracted and the real-time RT-PCR of IR and GLUT4 mRNAs was performed. As shown in Figure 2, the IR mRNA levels of C2C12 cells were increased by each of the treatments compared to the control group, the maximal effect observed with the OM2-treated group where IR mRNA level increased to about 125% of that of control group (Figure 2A). Similarly, the mRNA expression of GLUT4, the insulin-dependent glucose transporter, was also increased by each of the treatments compared to the control group (Figure 2B). In conclusion, all oligosaccharides OM, OM2 and OM4 enhanced the mRNA expression levels of IR and GLUT4 in C2C12 cells.

### Activation of PI3K/Akt pathway by oligomannuronate and its chromium (III) complexes

It is well known that insulin plays a central role in glucose homeostasis, and it accelerates glucose transport via activation of phosphatidylinositol 3-kinase (PI3K) and Akt, leading to glucose transporter 4 (GLUT4) translocation to the membrane in muscle cells [28], [29].

**Figure 3:**
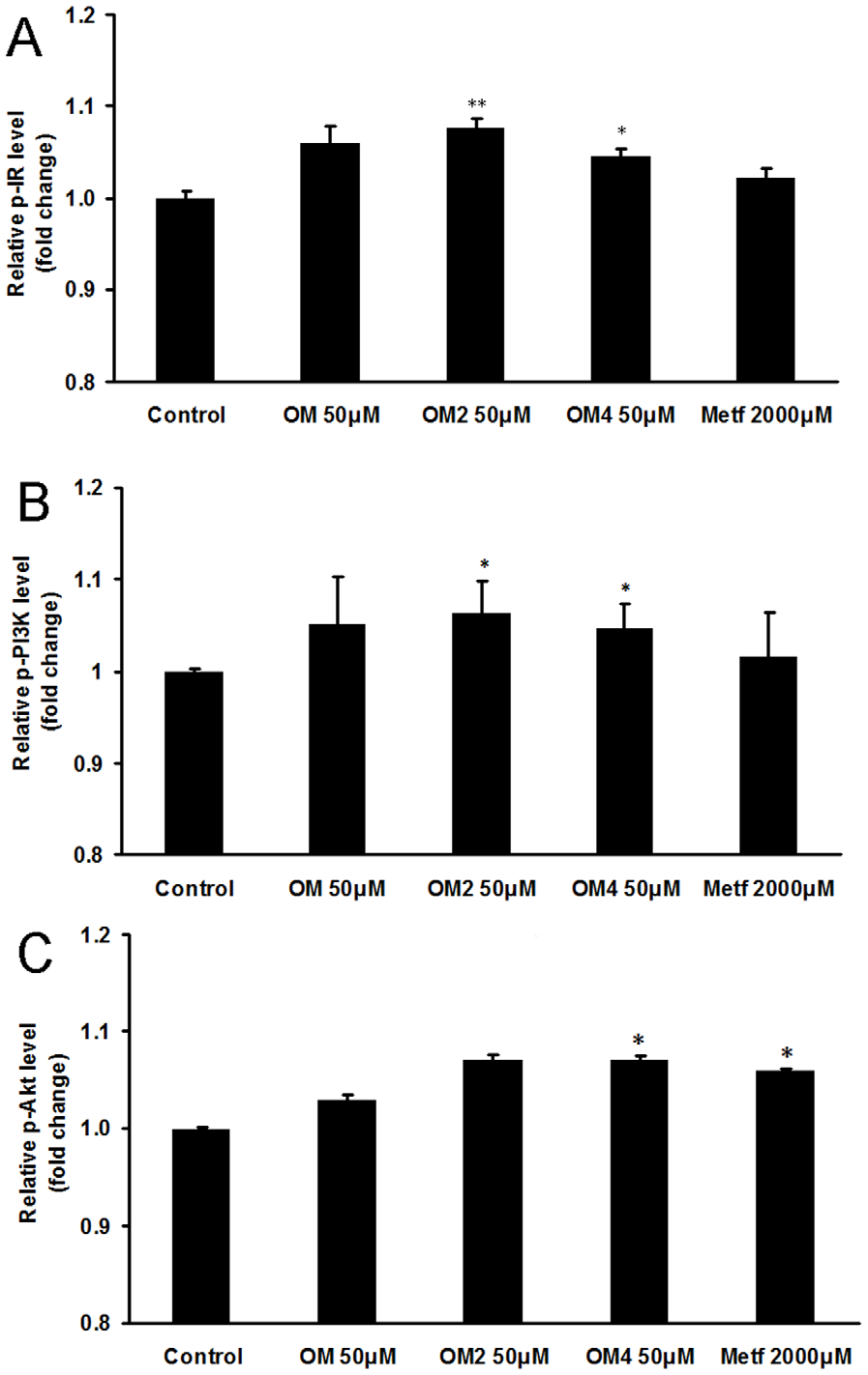
The influence of oligosaccharides on insulin signal pathway. C2C12 cells were treated with 50 µM oligomannuronate and its chromium (III) complexes (OM, OM2, OM4) or 2000 µM metformin at 37°C for 45 min. The cells were then lysed and the content of phosphorylated IR (A), PI3K (B) and Akt (C) in the cell lysates was evaluated by ELISA assay. Values are mean ± SD of the results from at least four independent experiments. Significance: *P<0.05, **P<0.01 versus controls without drug treatment.

Therefore we determined whether marine acidic oligosaccharide and its chromium (III) complexes could affect insulin signal pathway in C2C12 skeletal muscle cells. The C2C12 cells were treated with oligomannuronate and its chromium (III) complexes or metformin for 45 min, then the content of phosphorylated proteins in insulin pathway was measured by ELISA assay. As shown in Figure 3, the phosphorylated IR (p-IR) protein level increased significantly after the treatment of oligosaccharides at 50 µM compared to the non drug treated control group. Moreover, the phosphorylated PI3K (p-PI3K) and phosphorylated Akt (p-Akt) protein levels were also increased by marine oligosaccharides treatment (Figure 3). These results suggested that oligomannuronate and its chromium (III) complexes activated PI3K/Akt pathway in skeletal muscle cells, especially OM2.

### Activation of AMPK signaling pathway by oligomannuronate and its chromium (III) complexes

**Figure 4:**
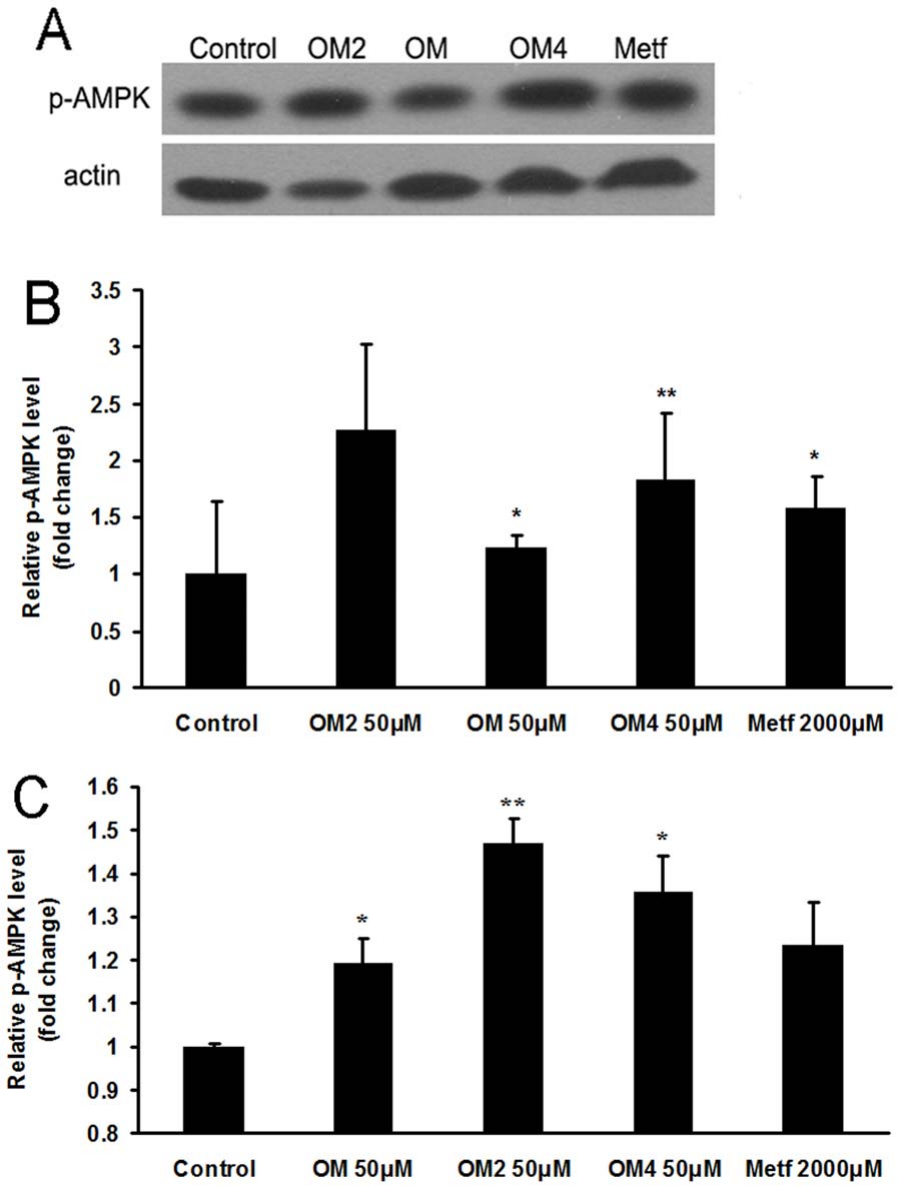
Effects of marine oligomannuronate and its derivatives on the production of phosphorylated AMPK (p-AMPK). (A)C2C12 cells were incubated with 50 µM oligomannuronate and its chromium (III) complexes (OM, OM2, OM4) or 2000 µM metformin at 37°C for 45 min. Then the cell lysates were separated by SDS-PAGE and blotted for p-AMPK expression by Western blotting. Blots were also probed for β-actin protein as loading controls. (B) Quantification of immunoblot for the ratio of p-AMPK protein to cellular β-actin. The ratio for non-drug treated cells (control) were assigned values of 1 and the results presented as mean ± SD (n = 3). (C) C2C12 cells were treated with 50 µM oligomannuronate and its chromium (III) complexes (OM, OM2, OM4) or 2000 µM metformin at 37°C for 45 min. The cells were then lysed and the content of phosphorylated AMPK in the cell lysates was evaluated by ELISA assay. Values are mean ± SD of the results from at least four independent experiments. Significance: *P<0.05, **P<0.01 versus controls without drug treatment.

The activation of AMPK was reported to be beneficial in ameliorating insulin resistance and type 2 diabetes [9], [30]. Therefore we determined whether marine acidic oligosaccharide and its chromium (III) complex derivatives could affect AMPK activity in skeletal muscle cells. The C2C12 cells were treated with oligomannuronate and its chromium (III) complexes or metformin for 45 min, then the content of phosphorylated AMPK (p-AMPK) protein was measured by western blot assay. As shown in Figure 4A, the three marine oligosaccharides all could increase the phosphorylation of AMPK when treated at the concentration of 50 µM. Moreover, the effects of OM2 and OM4 were even better than that of metformin (Figure 4B).

Furthermore, the results of ELISA assay also showed that the p-AMPK protein level increased dramatically after the treatment of oligomannuronate and its chromium (III) complexes at 50 µM compared to the non drug treated control group. Moreover, OM2 increased the production of p-AMPK to about 150% of that of control group, which as even better than the effect induced by metformin (120%) (Figure 4C). The result suggests that oligomannuronate and its chromium (III) complexes activated AMPK in skeletal muscle cells.

### Internalization of FITC-labeled oligomannuronate and its chromium (III) complex into skeletal muscle cells

**Figure 5:**
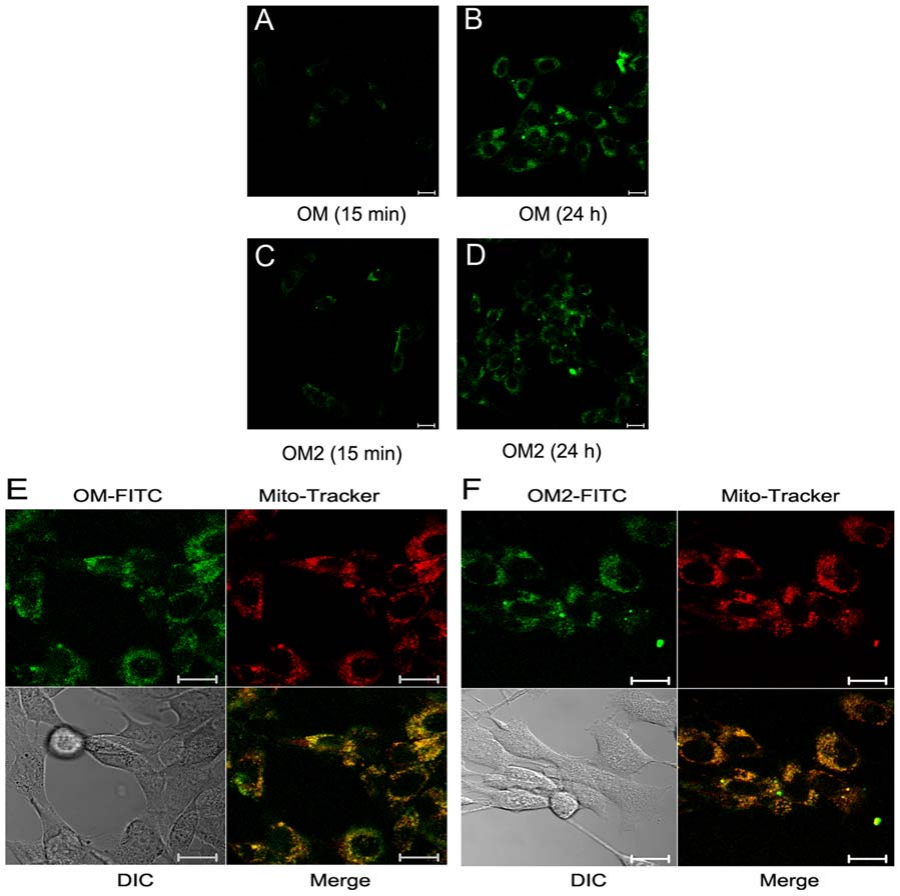
Internalization of FITC-labeled oligosaccharides. (A-B) C2C12 cells were incubated with FITC-labeled OM at 50 µM for 15 min and 24 h before fluorescence visualization. (C-D) C2C12 cells were incubated with FITC-labeled OM2 at 50 µM for 15 min and 24 h before fluorescence visualization with confocal microscopy. Scale bar represents 20 µm. (E-F) C2C12 cells were incubated with FITC-labeled OM (E) or OM2 (F) at 50 µM for 24 h before fluorescence visualization with confocal microscopy. The cells were also incubated with Mito-tracker for 30 min before imaging. Scale bar represents 20 µm.

FITC-labeled oligosaccharides (OM, OM2) were used to investigate whether they could enter into C2C12 cells or not. We found that the fluorescence intensity was increased with prolonged incubation of the cells with the oligosaccharides (Figure 5), indicated OM and OM2 are taken up by C2C12 cells. The internalization of oligosaccharides occurred within 15 min (Figure 5A and C), and more oligosaccharides were observed at 24 h (Figure 5B and D). These results suggested the internalization of oligomannuronate and its chromium (III) complex into C2C12 cells might be associated with the insulin-stimulated glucose uptake.

Furthermore, the exact intracellular distribution of OM and OM2 was also investigated by living cell imaging. After treated with FITC-labeled OM and OM2 for 24 h, the C2C12 cells were incubated with Mito-tracker to label mitochondria before imaging. As shown in Figure 5E and F,oligosaccharides OM and OM2 all were co-localized with mitochondria obviously after internalization into C2C12 cells,which suggested that these oligosaccharides could distribute to mitochondria in C2C12 cells. Taken together, these data indicated that the insulin sensitizing effects of marine oligosaccharides might be associated with the functions of mitochondria after internalization into skeletal muscle cells.

### Effects on energy metabolism of oligomannuronate and its chromium (III) complexes

**Figure 6:**
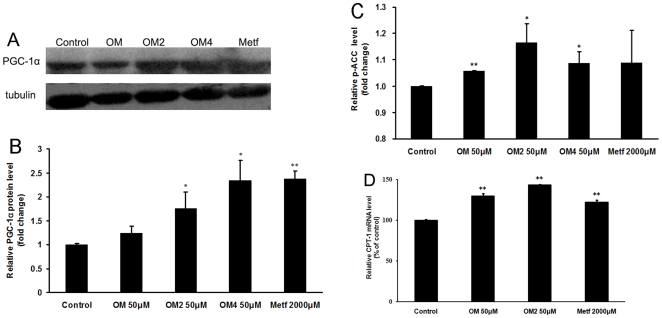
The influence of oligosaccharides on energy metabolism in C2C12 cells. (A) C2C12 cells were incubated with 50 µM oligomannuronate and its chromium (III) complexes (OM, OM2, OM4) or 2000 µM metformin at 37°C for 45 min. Then the cell lysates were separated by SDS-PAGE and blotted for PGC-1α expression by western blot analysis. Blots were also probed for α-tubulin protein as loading controls. (B) Quantification of immunoblot for the ratio of PGC-1α protein to cellular α-tubulin. The ratio for non-drug treated cells (control) were assigned values of 1 and the results presented as mean ± SD (n = 3). (C) C2C12 cells were treated with 50 µM oligomannuronate and its chromium (III) complexes (OM, OM2, OM4) or 2000 µM metformin at 37°C for 45 min. The cells were then lysed and the content of phosphorylated ACC in the cell lysates was evaluated by ELISA assay. Values are mean ± SD of the results from at least four independent experiments. (D) C2C12 cells were left untreated or treated with 50 µM oligomannuronate and its chromium (III) complexes (OM, OM2) or 2000 µM metformin in DMEM media for 24 hours. The mRNA levels of CPT-1 were analyzed by Real time RT-PCR as described in the text. The mRNA levels for untreated cells (control) were assigned values of 100. Values are mean ± SD (n = 4). Significance: *P<0.05, **P<0.01 versus controls without drug treatment.

The above results indicated that marine oligosaccharides were co-localized with mitochondria and enhanced the phosphorylation of AMPK, so we investigated the effects of oligosaccharides on cellular energy metabolism by evaluating the expression of PGC-1α in C2C12 cells. As shown in Figure 6A, the oligosaccharides OM2 and OM4 all significantly increased the production of PGC-1α, especially for OM4 which increased the expression of PGC-1α to about two times more than that in non drug treated control cells (Figure 6B). Moreover, the oligosaccharides treatment also enhanced the phosphorylation of ACC protein and increasd the mRNA expression of CPT-1, which suggested that these oligosaccharides could enhance the oxidation of fatty acid in C2C12 cells (Figure 6C and D). Taken together, the marine oligosaccharides could regulate the energy metabolism to attenuate insulin resistance.

## Discussion

Recently some reports indicated that marine derived polysaccharides can stimulate the insulin secretion in vitro, especially for the low molecular weight oligosaccharides around 3 kDa [31]. In this study, we investigated the insulin sensitizing effects and mechanisms of the marine acidic oligosaccharide and its chromium (III) complexes (∼3 kDa) in skeletal muscle cells. The results showed that both the marine acidic oligosaccharide and its chromium (III) complexes significantly enhanced insulin-stimulated glucose uptake in C2C12 cells. The oligomannuronate-chromium (III) complexes (OM2 and OM4) had better effect than the original oligosaccharide OM, especially for OM2 that contains 2% (w/w) chromium (III) in the oligosaccharide. It was reported that complexes of chromium (III) with organic ligands rather than in the form of inorganic salt generally showed low toxicities [32], which was also verified in this experiment (Figure 1A). Moreover, the most effective concentration of oligomannuronate and its chromium (III) complexes to improve glucose uptake was 50 µM, which is much lower than that of metformin (2000 µM).

In muscle cells, there are two important signal pathways to regulate glucose transport and metabolism, the insulin signaling pathway [33] and AMPK pathway [34]. Insulin signaling is mediated by cascades of phosphorylation/dephosphorylation events. Insulin signal transduction in skeletal muscle is mediated by a series of phosphorylation cascades linking initial activation of the insulin receptor (IR), a tyrosine kinase receptor, to downstream substrates [35]. Extensive studies have indicated that the ability of the receptor to autophosphorylate and phosphorylate intracellular substrates is essential for its mediation of the complex cellular responses to insulin [36]. IR plays an important role in the regulation of whole body metabolism and pathogenesis of diabetes. In the present study, we evaluated the mRNA expression of IR by quantitative RT-PCR analysis and the protein level of p-IR by ELISA assay, respectively. As we supposed, the mRNA and protein levels of IR in OM and OM2 treated groups dramatically increased compared to that in control group (Figure 2 and Figure 3), contributing to the improved insulin sensitivity. Activated IR transduces the insulin signal by activating PI3K/Akt pathway to promote glucose uptake. Current research seeks to ameliorate insulin resistance by finding ways to increase PI3K/Akt activity and restore insulin sensitivity [37], [38]. Activated Akt phosphorylates and regulates the activities of many downstream proteins involved in multiple aspects of cellular physiology. Here, ELISA results of p-PI3K and p-Akt indicated that the oligomannuronate and its chromium (III) complexes stimulated the activation of proteins in the PI3K/Akt signaling pathway, to an extent similar with insulin, and had an effect on glucose uptake. The PI3K/Akt pathway has been demonstrated to be able to regulate GLUT4 translocation. The importance of GLUT4 in glucose homeostasis has been studied extensively in recent years. GLUT4-mediated glucose transport in muscle is essential to the maintenance of glucose homeostasis [39]. Results indicated that the mRNA expression of GLUT4 increased in C2C12 cells after oligosaccharides treatments (Figure 2). Moreover, the increased production of GLUT4 might directly enhance the insulin stimulated glucose uptake. So the oligomannuronate and its chromium (III) complexes might be able to upregulate the insulin signaling to promote glucose transport through the PI3K/Akt pathway after internalization.

AMPK is considered a promising drug target for type 2 diabetes [40]. Activation of the enzyme in the liver or skeletal muscle with the cell-permanent AMP analog AICAR is associated with diminished gluconeogenesis [41] and enhanced glucose uptake [42], respectively. In skeletal muscle, AMPK activation may be involved in the effects of repeated exercise to improve insulin-sensitive glucose uptake, because of its ability to increase expression of GLUT4 and perhaps other effects [43]. In this study, we evaluated the effects of oligomannuronate and its chromium (III) complexes on AMPK activation in C2C12 cells by assessing the phosphorylation state of AMPK using western blot and ELISA assay. The treatment of oligomannuronate and its chromium (III) complex significantly increased the production of p-AMPK in C2C12 cells, and OM2 had better effect than the other two oligosaccharides and metformin. Combined with the upregulation of IR and GLUT4 mRNA expression, the increased production of phosphorylated AMPK by oligomannuronate and its chromium (III) complexes enhanced the GLUT4 expression to improve the glucose uptake. Further studies need to be carried out to decipher the intracellular targets of oligomannuronate and its chromium (III) complex both in vitro and in vivo.

A published report showed that polysaccharides could enter into liver cells by receptor-mediated endocytosis (RME) [44]. We found FITC-labeled OM and OM2 could enter into the C2C12 cells (Figure 5) within 15 min. The molecular mechanism of OM and OM2 internalization and its association with insulin related signaling pathway will be an interesting future research subject. Moreover, these two oligosaccharides distributed to mitochondria after internalization into C2C12 cells. These results suggested that the insulin sensitizing effects of marine oligosaccharides might be associated with the functions of mitochondria in skeletal muscle cells.

Insulin resistance was reported to be associated with impaired skeletal muscle oxidation capacity and reduced mitochondrial number and function [45]. AMPK increases GLUT4 expression by a PGC-1α-dependent pathway [46], [47]. Here we showed that the oligosaccharides significantly increased the production of PGC-1α, and enhanced the phosphorylation of ACC protein, which suggested that these oligosaccharides could enhance the fatty acid oxidation in skeletal muscle cells. Combined with the result that the oligosaccharides distributed to the mitochondria, we suppose that these oligosaccharides could improve the functions of mitochondria to attenuate the insulin resistance by regulating energy metabolism.

Chromium (III) is a cofactor for insulin function that increases insulin binding [48], the number of insulin receptors [49], and insulin receptor phosphorylation [50], resulting in enhanced glucose transport into liver, muscle, and adipose tissue. Furthermore, it was suggested that Chromium (III), like insulin, affects protein phosphorylation-dephosphorylation reactions [51]. The IR tyrosine kinase, responsible for the phosphorylation, can be activated by Chromium (III), to increase insulin sensitivity [52]. Moreover, chromium picolinate was reported to activate AMPK signaling pathway in cardiac and skeletal muscle [53]. Here we showed that the oligomannuronate-Chromium (III) complex OM2 had a better effect on increasing insulin sensitivity than the original oligosaccharide OM, which suggested the introduction of Chromium (III) to the oligosaccharide might be able to increase the phosphorylation of AMPK and PI3K in the signaling pathway. However, the insulin sensitizing effect of OM4 was lower than that of OM2 although it had higher content of chromium (III) than OM2, which indicates that the content of chromium is not the major reason for the observed insulin sensitizing effect and the oligomannuronate-Chromium (III) complex OM2 itself has best insulin sensitizing effect.In conclusion, we found that oligomannuronate and its chromium (III) complexes, which were less cytotoxic than metformin, enhanced glucose uptake in C2C12 cells. The improvement of insulin sensitivity might be attributed to upregulation of the expression of IR and GLUT4 by activating both insulin signal (PI3K/Akt) and AMPK signal pathways in skeletal muscle. Moreover, oligosaccharides distributed to the mitochondria in C2C12 cells and increased the expression of PGC-1α, which suggested that the actions of these oligosaccharides might be associated with mitochondria. Furthermore, introduction of Chromium (III) to the marine oligosaccharide increased its bioactivity to some extent. Therefore the oligomannuronate-chromium (III) complex could be considered a potential agent in the treatment of type 2 diabetes due to its activation of PI3K/Akt and AMPK. This is the first report to suggest a possible mechanism by which the oligomannuronate-chromium (III) complex improves insulin sensitivity. We conclude that the oligomannuronate-chromium (III) complex might provide the basis for an adjuvant therapy of type 2 diabetes by enhancing insulin sensitivity with a lower toxicity profile than that of metformin.

## Materials and Methods

### Materials

The marine-derived oligomannuronate and its chromium (III) complexes named OM, OM2 and OM4 were provided by Glycoscience and Glycoengineering Laboratory, school of Medicine and Pharmacy, Ocean University of China. The molecular masses of OM, OM2 and OM4, measured by high performance gel permeation chromatography, were 2.8, 3.0 and 3.2 kDa, respectively.

Bovine insulin was purchased from Calbiochem (USA). [^3^H]-2-deoxy-D-glucose was purchased from Sigma Chemical Company (USA). Other reagents were obtained from Sigma Chemical Company (USA). Anti-Phospho-AMPKα (Thr172) and Anti-PGC-1α antibodies were from Cell signaling Technology, Inc (USA). Anti-β-actin and anti-α-tubulin antibodies were obtained from Santa Cruze Biotechnology, Inc (USA). HRP-linked secondary antibodies were purchased from Cell signaling Technology, Inc (USA).

### Cell culture

The C2C12 myoblast cell line from mouse skeletal muscle was purchased from the ATCC Global Bioresource Center (USA). Cells were maintained in Dulbecco's Modified Eagle's medium (DMEM, GibcoBRL, USA) supplemented with 10% fetal bovine serum (FBS) (GibcoBRL), and containing 4.5 g/L glucose, 25 mM HEPES, 100 U/ml penicillin, and 100 µg/ml streptomycin at 37°C in a humidified 95% air and 5% CO_2_ atmosphere.

### Cytotoxicity assay

The cytotoxicity of compounds was measured by the MTT (3-[4, 5-dimethylthiazol-2-yl]-2, 5-diphenyl tetrazolium bromide; Sigma-Aldrich, USA) assay. Confluent C2C12 cell cultures in 96-well plates were exposed to different concentrations of compounds in triplicate for 24 h at 37°C under 5% CO_2_ atmosphere. Next, 20 µL of PBS containing MTT (final concentration: 0.5 mg/mL) was added to each well. After 4 h incubation at 37°C, the supernatant was removed and 200 µL of DMSO was added to each well to solubilize the formazan crystals. After vigorous shaking, absorbance values were measured in a microplate reader (Bio-Rad, USA) at 570 nm.

### Insulin-stimulated glucose transport assay

Briefly, C2C12 myoblast cells were incubated with various concentrations of indicated drugs for 24 hours in culture medium. After treatment, cells were washed three times with PBS, and then incubated with or without 100 nM insulin in the KRP buffer for 30 minutes, and the assay was initiated via the addition of nonmetabolizable glucose analog [^3^H]-2-deoxyglucose (finally 0.5 U Ci/mL) to each of the wells for 10 minutes at 37°C. The assay was terminated by the addition and subsequent washing of the cells with ice-cold PBS. The cells were then lysed in 100 mM NaOH. Radioactivity was evaluated via scintillation counting of the lysates, while total protein contents were determined by the Bradford procedure (Bio-Rad Laboratory, Richmond, CA, USA). The values were corrected for non-specific glucose uptake (the cytochalasinB cpm (counts per minute) values). The results were expressed as fold stimulation of the controls.

### Real-time RT-PCR assay

After incubation with different concentrations of oligosaccharides for 24 h, C2C12 cells were washed twice with ice-cold PBS. Total RNA was isolated using the TRIzol reagent (Invitrogen, USA) and 1 µg RNA was reverse-transcribed into cDNA using PrimeScript® One Step RT-PCR Kit (Takara, Japan). The following primer pairs were used as reported previously [45]:

Insulin receptor (IR): Forward:5′-AATGGCAACATCACACACTACC-3′, Reverse: 5′-CAGCCCTTTGAGACAATAATCC-3′; Glucose transporter 4 (GLUT4): Forward: 5′-CAACGTGGCTGGGTAGGCAAGGT-3′, Reverse: 5′-CGGAGAGAGCCCAGAGCGTAG TA-3′; Carnitine palmitoyltransferase 1 (CPT-1): Forward: 5′-CGTGACGTTGGACAGATC-3′, Reverse: 5′-TCTGCGTTTATGCCTATC-3′; 18S rRNA: Forward: 5′-AGGAAGTCCCTCACC CTCCCAAAA-3′, Reverse: 5′-CAGAAGCAATGCTGTCACCTTCCC-3′. Target cDNA levels were quantified by RT-PCR using the ABI PRISM 7500 sequence detection system (Applied Biosystems, USA) utilizing SYBR green. All reactions were performed in triplicate. The mouse 18S rRNA gene served as the endogenous reference gene. The evaluation of relative differences of PCR product among the treatment groups was carried out using the ΔΔCT method. The reciprocal of 2CT (using CT as a base 2 exponent) for each target gene was normalized to that for 18S rRNA, followed by comparison with the relative value in control cells. Final results are presented as a percentage of control.

### Living cell imaging by confocal microscopy

The oligomannuronate and its chromium (III) complex (OM and OM2) were labeled by fluorescein isothiocyanate (FITC) as described before [44]. C2C12 cells grown on glass coverslips overnight were first treated with FITC-labeled OM and OM2 for 15 min and 24 h at their maximum efficient concentration (50 µM). The cells were then washed four times with ice-cold PBS (pH 7.4). For co-localization assay, the cells were incubated with Mito-tracker for 30 min before imaging. The green fluorescence of FITC-OM and FITC-OM2 was measured at 520±20 nm by Laser Scanning Confocal Microscope (Zeiss LSM 510, GER).

### ELISA assay

The C2C12 cells (5×10^5^ cells per well) were trypsinized and then transferred to a 6-well culture plate containing 2 mL DMEM-FBS (10%) solution. After incubation for 24 h, 50 µM of oligomannuronate-chromium (III) complex (OM, OM2, OM4) or 2000 µM metformin were separately added and incubated for 45 min at 37°C. At last, C2C12 cells were rinsed twice with PBS, and then treated with 100 µL of lysis buffer (Beyotime Biotechnology, China) for 30 min on ice. The lysates were collected and centrifuged (12000 g; 5 min; 4°C), and the protein content was measured using BCA assay. The ELISA assays were performed according to the manufacturer's protocol of the p-IR, p-PI3K, p-Akt, p-AMPK, and p-ACC assay kit, respectively (Jingtian Biotech, Shanghai, China).

### Western blot assay

As previously described [54], C2C12 cells were rinsed twice with ice-cold PBS after drug treatment for 45 min, and then scraped into with ice-cold 100 µL of lysis buffer (20 mM Tris pH 7.5, 150 mM NaCl, 1% Triton X-100, 1 mM sodium pyrophosphate, 1 mM EDTA, 1 mM Na_3_VO_4_, 1 µg/mL leupeptin, 1 mM phenylmethylsulphonyl fluoride) for 30 min on ice. After vortex-mixed and centrifuged (12000 g; 5 min; 4°C), the protein content was measured using BCA assay. Total protein extracts (50–80 µg) were subjected to SDS-PAGE on 10% polyacrylamide gels and transferred to nitrocellulose (NC) membranes. After transferring, the filters were blocked with 5% non-fat dry milk in TBS containing 0.1% Tween 20 for 1.5 h at room temperature, and subsequently subjected to immunoblot analysis by incubation with primary antibodies overnight at 4°C: phospho-AMPK-α (Thr172) (1∶1000 dilutions), PGC-1α (1∶1000 dilutions), β-actin (1∶5000 dilutions), α-tubulin (1∶5000 dilutions). The NC filters were then washed three times for 15 min each time with TBST followed by 1 h of incubation with secondary antibody conjugated to horseradish (1∶5000 dilutions) (Cell Signaling Technology, Beverly, MA). After that, the filters were washed three times and subsequently exposed to an enhanced chemiluminescence detection (Super Signal* West Pico, Thermo Scientific, USA).

### Statistical analysis

All experiments were performed in triplicate. Data were presented as mean ± S.D. Statistical significance was evaluated by unpaired Student's t-test for comparison of means. Differences were considered statistically significant at P<0.05.
